# Cluster randomised comparison of the effectiveness of 100% oxygen versus titrated oxygen in patients with a sustained return of spontaneous circulation following out of hospital cardiac arrest: a feasibility study. PROXY: post ROSC OXYgenation study

**DOI:** 10.1186/s12873-018-0214-1

**Published:** 2019-01-25

**Authors:** Matthew Thomas, Sarah Voss, Jonathan Benger, Kim Kirby, Jerry P. Nolan

**Affiliations:** 1 Intensive Care Unit, University Hospitals Bristol NHS Foundation Trust Bristol Royal Infirmary, Bristol, BS2 8HW England; 2 Department of Emergency Care, University of West of England Glenside Campus (1H14), Blackberry Hill, Bristol, BS16 1DD England; 3 Academic Department of Emergency Care, University Hospitals Bristol NHS Foundation Trust Emergency, Bristol, England; 4 Emergency Department Bristol Royal Infirmary, University Hospitals Bristol NHS Foundation Trust Emergency, Bristol, BS2 8HW England; 5 Consultant in Anaesthesia and Critical Care Department of Anaesthesia, Royal United Hospital Bath NHS Trust, Bath, BA1 3NG England

## Abstract

**Background:**

Hyperoxia following out of hospital cardiac arrest (OHCA) is associated with a poor outcome. Animal data suggest the first hour post resuscitation may be the most important. In the UK the first hour usually occurs in the prehospital environment.

**Methods:**

A prospective controlled trial, cluster randomised by paramedic, comparing titrated oxygen with 100% oxygen for the first hour after return of spontaneous circulation (ROSC) following OHCA.

The trial was done in a single emergency medical services (EMS) system in the United Kingdom (UK) admitting patients to three emergency departments. This was a feasibility trial to determine whether EMS staff (UK paramedics) can be successfully recruited and deliver the intervention.

**Results:**

One hundred and fifty seven paramedics were approached and 46 (29%) were consented, randomised and trained. During the study period 624 patients received a resuscitation attempt. A study paramedic was in attendance at 73 (12%) of these active resuscitations. Thirty-five patients were recruited to the trial, 32 (91%) were transported to hospital and 13 (37%) survived to 90 days. The intervention was initiated in 27/35 (77%) of enrolled patients. A reliable oxygen saturation trace was obtained in 22/35 (69%) of patients. Data collection was complete in 33/35 (94%) of patients.

**Conclusions:**

It may be feasible to complete a randomised trial of titrated versus unrestricted oxygen in the first hour after ROSC following OHCA in the UK. However, the relatively few eligible patients and incomplete initiation of the allocated intervention are challenges to future research.

**Trial registration:**

ISRCTN 49548506 retrospectively registered on 24.11.2016.

## Background

Survival rates following out of hospital cardiac arrest (OHCA) are disappointingly low: analysis of 70 studies documented survival to hospital discharge in just 7.6% (95% confidence interval (CI) 6.7–8.4) [1]. Death on the intensive care unit (ICU) is usually caused by withdrawal of life-sustaining treatment (WLST) because of perceived severe neurological injury [2]

Although the delivery of oxygen to tissues is essential for human life, there is evidence that high oxygen concentrations after return of spontaneous circulation (ROSC) can be harmful. This is probably due to the many reactive oxygen species that are created during reperfusion [3]. Administration of high concentrations of oxygen has been shown to be harmful in stroke, myocardial infarction and in neonatal resuscitation [4–6] Animal data indicate that avoidance of hyperoxia, particularly during the first hour after ROSC, reduces brain injury [7].

Large human registry studies have demonstrated that normoxia in the first 24 h following a cardiac arrest is associated with improved outcome compared with either hyperoxia or hypoxia, [8] whilst animal data suggest that most benefit from the prevention of hyperoxia is gained during the first hour following ROSC [9]. One small clinical study that compared administration of 30% oxygen with 100% oxygen after OHCA showed lower neuron specific enolase (NSE – a marker of neurological injury) values in one subgroup of patients who were allocated to 30% oxygen and not cooled [10].

In most emergency medical services (EMS) systems, the first hour following ROSC will be prehospital. We therefore designed a trial to examine the feasibility of administering titrated oxygen versus 100% oxygen for the first hour following ROSC to see if paramedics are able to safely titrate oxygen and measure corresponding oxygen saturations.

Our aims were to assess the feasibility of completing a cluster-randomised clinical trial to determine if titrated oxygen therapy (target blood oxygen saturation by pulse oximetry (SpO_2_) 94–98%) for 1 h after ROSC improves outcome compared with the use of 100% oxygen during this hour.

## Methods

### Design

Prospective controlled trial, cluster randomised by paramedic, comparing titrated oxygen with 100% oxygen for the first hour after ROSC following OHCA. Ethics committee approval was obtained from the National Research Ethics Service Committee; South Central - Oxford C (14/SC/1269).

### Setting and population

North Sector of the South Western Ambulance Service, in the cities of Bristol and Bath, a predominantly urban area in the United Kingdom (UK) with a population of 1 million. The ambulance service is delivered primarily by paramedics supported by a few voluntary doctors, but there is no 24 h cover by doctors. Paramedics were approached via ambulance service bulletins and personal letter and invited to participate. There were no additional requirements for the paramedics. Patients were enrolled if they were 18 years or older and had an OHCA that was not caused by trauma (trauma included drowning and hanging).

### Randomisation

The unit of randomisation was the paramedic, and not the patient. Individual paramedics were allocated randomly to one of two groups in a 1:1 ratio. This cluster randomisation by paramedic was also used in a recently completed study of airway interventions in out of hospital cardiac arrest [11].

Randomisation was performed using a method with secure allocation concealment that cannot be changed once allocated. The allocation was stratified by prior experience (more or less than 4 years full-time equivalent experience as an operational “front line” paramedic), to ensure that the experience of attending staff was equally distributed between the two trial groups. The sequence of random allocations was generated by computer and concealed until a paramedic had been recruited. This randomization process was carried out independently by the Bristol Clinical Trials and Evaluation Unit.

### Treatment protocol

Paramedics who agreed to take part attended a training session, and were then given the opportunity to consent to take part in the trial. The training was delivered by an experienced research paramedic and a doctor with experience of prehospital care and expertise in ventilation. The primary focus of the training was to explain the treatment algorithm and how to adhere to it. It also included research ethics, good clinical practice and completion of case record forms.

**Fig. 1:**
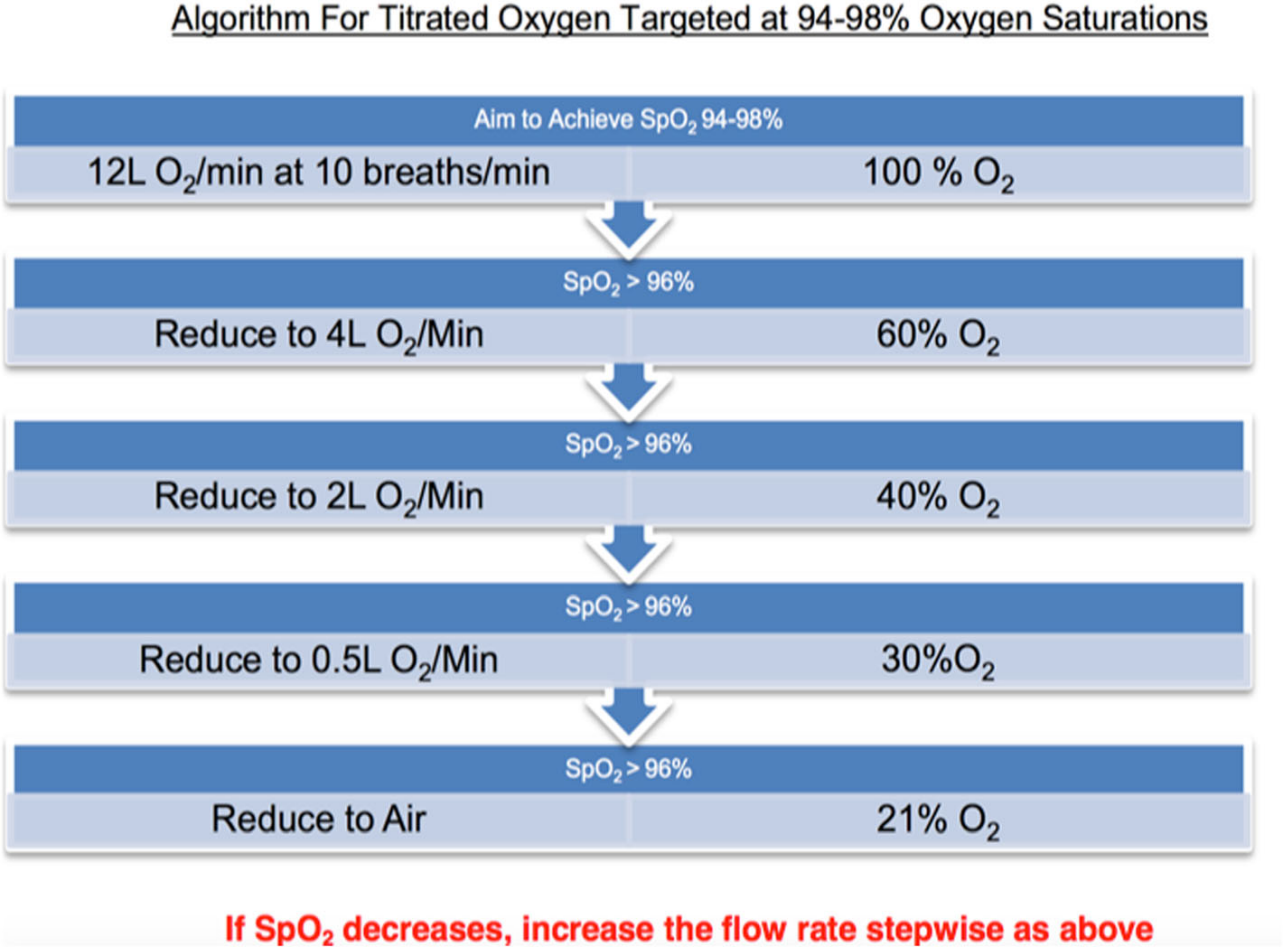
Titrated Oxygen Protocol

The treatment algorithm for the titrated oxygen arm is shown in Fig. 1. Paramedics were advised to consider titration of oxygen every 2 min. If there was no reliable saturation trace, or oxygen saturations fell below 94% study paramedics increased oxygen delivery in a stepwise approach as detailed in Fig. 1.

Paramedics in the 100% oxygen arm used 100% oxygen throughout the first hour following ROSC. All other elements of routine care were provided as usual.

Patients received the allocated treatment for one hour following ROSC. If the patient was admitted to the emergency department during the first hour the allocated treatment was continued by emergency department staff. After one hour, usual care was provided, including oxygen delivery at the discretion of the treating clinician.

### Outcomes

The primary outcomes for this feasibility study were:

1. Proportion of eligible paramedics attending training and consenting to take part.
2. Proportion of eligible patients enrolled in the study.
3. Proportion of enrolled patients in whom the intervention was initiated (defined as obtaining a reliable SpO_2_ reading immediately after sustained ROSC).
4. Proportion of enrolled patients in whom the assigned oxygen therapy was continued for the duration of transport in an ambulance or up to 60 min.
5. Proportion of enrolled patients handed over to emergency department care in whom the assigned oxygen therapy was continued from arrival at hospital, up to 60 min.
6. Completeness of required data collection.

The secondary outcomes (collected by postal contact and questionnaire) were:

1. Proportion of surviving participants providing quality of life data at discharge and 90 days.

## Survival to discharge and 90 days

### Analysis

Trial data are reported on an intention-to-treat basis. Binary outcomes are reported as number and percentage, and continuous outcomes are reported as mean and standard deviations or median and interquartile ranges as appropriate. Formal comparisons between the groups were not made as this feasibility study was not powered for comparative analysis.

## Results

### Paramedic recruitment

One hundred and fifty-seven paramedics were approached over a three-month period and 46 (29%) provided consent and underwent, randomisation and training. Three paramedics (7%) withdrew during data collection because of changes in their employment circumstances.

### Number of OHCA during the study period

**Fig. 2:**
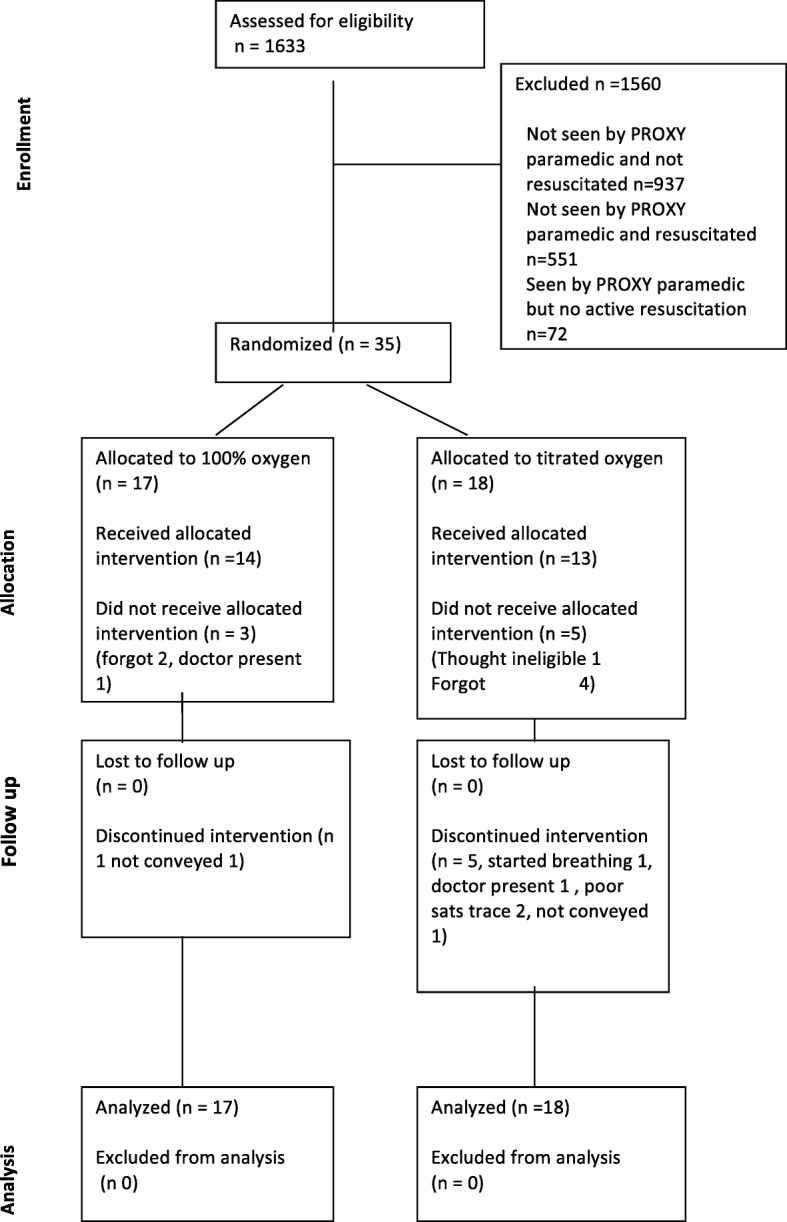
Trial flow (Consort) diagram

Study paramedics enrolled patients over 6 months. During this time 1633 OHCAs were identified from ambulance service data, with 624 (38%) undergoing an active resuscitation attempt (Fig. 2). A study paramedic was in attendance at 73 (12%) of these active resuscitations.

### Study patients

Thirty-five patients were enrolled and included in the final analysis. Enrolment was evenly divided across the study arms and the arrest characteristics were similar (Table 1). The methods of airway management used during the resuscitation and the airway management in place at identified time points in the patient’s care are described in Table 2. Of the 35 patients, 32 (91%) were transported to hospital, 13 (37%) survived to discharge and 13 (37%) survived to 90 days (Table 3).

**Table 1:** Patient and cardiac arrest characteristics by trial arm (paramedic reported)

		Arm A 100% oxygen (*n* = 17)	Arm B titrated oxygen (*n* = 18)	Total (*n* = 35)
Sex	Female n (%)	5 (29)	5 (28)	10
	Male n (%)	12 (71)	13 (72)	25
Age (mean, years)		64	70	67
Witnessed	No n(%)	3 (18)	1 (6)	4
	Yes n(%)	14 (82)	17 (94)	31
EMS witnessed	No n(%)	15 (88)	9 (50)	24
	Yes n(%)	2 (12)	9 (50)	11
Bystander cardiopulmonary resuscitation	EMS witnessed n(%)	2 (12)	9 (50)	11
	No n(%)	4 (24)	4 (22)	8
	Yes n(%)	11 (64)	5 (28)	16
First monitored rhythm	Shockable n (%)	6 (35)	10 (56)	16
	Non shockable n (%)	10 (59)	6 (33)	16
	Missing n (%)	1 (6)	2 (11)	3

*EMS* Emergency Medical Services

**Table 2:** Methods of airway management used during patient care

Method of airway management used - Arm A - 100% oxygen (*n* = 17)					
	Tracheal tube	Supraglottic airway device	Oropharyngeal airway	Other	Missing
On loading into the ambulance n (%)	6 (35)	4 (24)	2 (12)	2 (12)	1 (6)
En route to ED n (%)	0	0	0	1 (6)	14 (82)
On arrival at ED n (%)	8 (47	1 (6)	1 (6)	2 (12)	3 (18)
Not Conveyed	2 patients				
Method of airway management used - Arm B - Titrated oxygen (*n* = 18)					
On loading into the ambulance n (%)	3 (17)	3 (17)	3 (17)	6 (33)	2 (11)
En Route to ED n (%)	0	0	1 (6)	1 (6)	15 (83)
On arrival at ED n (%)	5 (28)	1 (6)	1 (6)	6 (33)	4 (22)
Not Conveyed	1 patient				

*ED* Emergency Department

**Table 3:** Patient outcomes and follow up

	Arm A 100% oxygen (*n* = 17)	Arm B titrated oxygen (*N* = 18)	Total (*n* = 35)
Re-arrest following initial ROSC	8 (47)	3 (17)	
Death recognised on scene n (%)*	2 (12)	1 (6)	3 (18)
Survival to discharge n (%)	3 (18)	10 (55)	13 (37)
Survival to 90 days n (%)	3 (18)	10 (55)	13 (37)

^*^Data missing for two patients; one from each group

### Intervention success

Baseline characteristics were similar in both arms (Table 1) with the exception of EMS witnessed. The intervention was initiated in 27 of 35 (77%) of enrolled patients; 82% in the control arm and 72% in the intervention arm. A reliable SpO_2_ reading was reported in: 22 (69%) of patients immediately after sustained ROSC; 19 (66%) of patients on loading into the ambulance; 21 (70%) of patients en route to hospital; 20 (69%) of patients on arrival at the receiving hospital (percentages calculated only on those patients transported, and for whom data were available).

Where the intervention was initiated it was continued for the full 60 min during the pre-hospital phase of care in 16/27 (59%) of cases. In 10 cases where it was not, the intervention was discontinued in the pre-hospital phase for 6 patients (three not conveyed, two unable to measure SpO_2_, one for other reasons) and continued in the ED for four patients.

Arterial blood gas samples were taken on six patients in the titrated oxygen group with a mean PaO_2_ of 13.5 kPa(StD 9.2) and eight in the 100% oxygen group with a mean PaO_2_ of 15.4 kPa.(StD 14.7).

Case report form data were collected for 33/35 (94%) of patients and contained > 90% complete data.

Blood oxygen saturation values by pulse oximetry and oxygen flow rates are shown in Tables 4 and 5. Three patients in the 100% group and four in the titrated oxygen group had SpO_2_ values documented below 90% at any point.

**Table 4:** Oxygen saturations recorded during patient care

Oxygen saturations recorded- Arm A - 100% oxygen (*n* = 17)				
	SpO_2_ ≤ 94%	SpO_2_ > 94%	Missing Data	Unable to Measure
On loading into ambulance n (%)	3 (18)	6 (35)	2 (12)	4 (24)
En Route to ED n (%)	3 (18)	7 (41)	1 (6)	4 (24)
On arrival at ED n (%)	2 (12)	7 (41)	2 (12)	4 (24)
Not Conveyed	2 Patients			
Oxygen saturations recorded - Arm B titrated oxygen (*n* = 18)				
On loading into ambulance n (%)	4 (22)	8 (44)	1 (6)	4 (22)
En Route to ED n (%)	5 (28)	8 (44)	1 (6)	3 (17)
On arrival to ED n (%)	5 (28)	8 (44)	1 (6)	3 (17)
Not Conveyed	1 Patient			

*ED* Emergency Department, *SpO*_*2*_ Oxygen saturation

**Table 5:** Oxygen flow rates – self-inflating bag ventilation

If self –inflating bag ventilation; flow rate l/min - Arm A 100% Oxygen (*n* = 17)					
	< 15 l/min	15 l/min	Mechanically Ventilated 100% oxygen	Missing Data	Nil – maintaining SpO_2_ values with air
On loading n (%)	1 (6) (10 l/min)	9 (53)	1 (6)	3 (18)	1 (6)
En Route to ED n (%)	0	6 (35)	5 (29)	3 (18)	1 (6)
On arrival at ED n (%)	0	6 (35)	5 (29)	3 (18)	1 (6)
Non Conveyed n (%)	2 Patients				
If self-inflating bag ventilation; flow rate l/min - Arm B Titrated Oxygen (n = 18)					
Flow Rate n (%)	< 15 l/min	15 l/min	Mechanically Ventilated 100% oxygen	Missing Data	Nil – maintaining SpO_2_ values with airown saturations
On loading n (%)	4 (22) (8–12 l/min)	5 (28)	2 (11)	3 (17)	3 (17)
En Route to ED n (%)	4 (22) 1–12 l/min)	3 (17)	2 (11)	2 (11)	3 (17)
On arrival at ED n (%)	3 (17) (12 l/min)	3 (17)	1 (6)	2 (11)	4 (22)
Non Conveyed	1 Patient				

*ED* Emergency Department

Oxygen flow rates were documented in 21/35 patients (60%). In the titrated oxygen group nine patients had reliable documentation with four patients having successful titration of oxygen below 15 l min^− 1^. For one patient the inspired oxygen concentration was reduced to 21% (air) on arrival in the emergency department.

### Clinical outcomes

Clinical outcomes are in Table 3, but we made no comparison between groups because of the feaibility design and small sample size. Chance differences in the patients enrolled will have affected the clinical outcome data significantly.

### Patient follow up at 90 days

Patients were contacted by post to complete quality of life (QoL) questionnaires where they survived to 90 days and consented to be contacted. Five patients survived to 90 days and also consented to complete QoL questionnaires. Three patients returned completed questionnaires to the study team.

## Discussion

### Summary of principal findings

- We have shown that amongst UK paramedics it is potentially feasible to perform a trial of titrated oxygen in the first hour following ROSC after OHCA.
- Recruitment of paramedics can be difficult in the UK setting.
- Accurate recording of SpO_2_ values is challenging or impossible in some patients, and measures must be taken to avoid hypoxia in these individuals.
- Paramedics are willing and able to follow an algorithm for oxygen titration.

In the intervention arm, paramedics were able to titrate the inspired oxygen concentration in 72% of eligible patients. These findings improve on a previous study to examine pre-hospital oxygen titration, which analysed data from 17 patients in New Zealand and concluded that safe delivery of titrated oxygen therapy in the pre-hospital period was not feasible [12]

Similar trials include a small pre-hospital feasibility study of 28 cardiac arrest patients who were allocated randomly to one of two groups immediately after ROSC [10]. Group A received 30% oxygen and group B received 100% oxygen. The study concluded that patients who received 30% oxygen had acceptable oxygen saturations (although the inspired oxygen concentration had to be increased in 5 of the 14 patients in the 30% group to maintain an SpO_2_ ≥ 95%). The study demonstrated feasibility, however a larger trial did not follow.

A second randomised trial conducted in Australia documented widely scattered blood oxygen saturation values that were downloaded from pulse oximeter monitors; consequently, the study was discontinued early [12]. Following the completion of our trial a further feasibility study has been published, [13] showing that a titrated oxygen strategy is possible. Arterial blood oxygen desaturations were more common in the titrated group, however the findings were used to support the case for a large randomised controlled trial. Our findings also suggest that oxygen saturation can be difficult to measure in the prehospital environment, and that arterial blood oxygen desaturations occur, though patient characteristics, paramedic training, organisation of emergency medical systems and distances travelled all differ between the UK and Australia.

### Limitations of the trial

We relied on paramedic reporting of pulse oximeter oxygen saturation readings and successful oxygen titration, rather than objective measures, since at the time of the trial automated recording of oxygen saturations was not available in our EMS. Also, we did not provide a standardised definition of ‘reliable’ pulse oximetry readings, and so there is likely to have been some variability in interpretation.

We used a cluster-randomised design by paramedic, which meant that the individuals delivering the intervention and reporting some of the outcomes were not blinded. Paramedics were not selected, but volunteered, and this may reduce the generalizability of our findings when applied to all paramedics. Poor recruitment of volunteer paramedics remains an issue for future trials. However, we required only a few individuals and so did not maximise our recruitment strategy; the same ambulance service recently recruited many more paramedics for a considerably larger randomised controlled trial [11]. In our opinion, individual patient randomisation and paramedic blinding are not practical within UK EMS systems during time critical interventions such as cardiac arrest, and cluster randomisation is the only feasible approach.

### Future trials

We have collected relevant data, however the required sample size for any future study will depend on the chosen primary outcome. The relatively few eligible patients, along with incomplete initiation of the intervention and some missing data elements, makes a highly clinically relevant outcome such as mortality very challenging to achieve. A more realistic approach might be to evaluate serum NSE values as a measure of neurological injury [10]. Accurate SpO_2_ measurement and automatic recording is also crucial to further trials in this area.

The inability to blind paramedics to the intervention is also a limitation. Future studies would benefit from a more objective and reliable method of measuring tissue and brain oxygenation, although this can be difficult in a prehospital environment [14].

## Conclusion

We have shown that it may be feasible to complete a randomised trial of titrated versus unrestricted oxygen in the first hour after ROSC following OHCA in the UK. However, the relatively few eligible patients and incomplete initiation of the allocated intervention are challenges to future research. More feasibility work is required to explore the best way of addressing this important research question.

## Abbreviations

ED: Emergency Department
EMS: Emergency Medical Services
NSE: Neuron specific enolase
OHCA: Out of hospital cardiac arrest
ROSC: Return of spontaneous circulation
UK: United Kingdom
